# Phylogenetic analysis and tissue distribution of elasmobranch glucose transporters and their response to feeding

**DOI:** 10.1242/bio.016709

**Published:** 2016-02-12

**Authors:** Courtney A. Deck, Christophe M. R. LeMoine, Patrick J. Walsh

**Affiliations:** 1 Department of Biology, University of Ottawa, Ottawa, Ontario K1N 6N5, Canada; 2 Bamfield Marine Sciences Centre, Bamfield, British Columbia V0R 1B0, Canada; 3 Department of Biology, Brandon University, Brandon, Manitoba R7A 6A9, Canada

**Keywords:** Elasmobranch, Holocephalan, Glucose transporter, Rectal gland

## Abstract

Elasmobranch diets consist of high quantities of protein and lipids, but very low levels of carbohydrates including glucose. Reflecting this diet, most tissues use lipids and ketone bodies as their main metabolic fuel. However, the rectal gland has been shown to be dependent on glucose as a fuel, so we hypothesized that glucose transporters (GLUTs) would be present and upregulated in the gland during times of activation (e.g. following a meal). In this study, we searched for and identified putative class I GLUTs in three elasmobranchs and a holocephalan using transcriptomes, and used these to reconstruct a Bayesian phylogeny. We determined that each of the four species possessed three of the four class I GLUT sequences, but the identities of the isoforms present in each species differed between the elasmobranchs (GLUT1, 3 and 4) and the holocephalan (GLUT1, 2 and 3). We then used qPCR to measure mRNA levels of these GLUTs in the rectal gland, liver, intestine, and muscle of fed and starved spiny dogfish (*Squalus suckleyi*). The rectal gland data showed higher mRNA levels of *GLUT4* in the starved relative to the fed fish. In the muscle, both *GLUT1* and *4* were significantly elevated at 24 h post-feeding, as was the case for *GLUT4* in the liver. In the intestine on the other hand, *GLUT4* was significantly elevated by 6 h post-feeding, remaining elevated through 48 h. We suggest that GLUT4 has taken on the role of GLUT2 in elasmobranchs as the expression patterns observed in the liver and intestine are representative of GLUT2 in other vertebrates.

## Abstract (precis)

**Summary:** Our results indicate the presence of three putative glucose transporters in elasmobranchs (GLUT1, 3, 4) and holocephalans (GLUT1, 2, 3). We determined that *GLUT1* and *GLUT4* mRNA levels change in various dogfish tissues in response to feeding.

## INTRODUCTION

Elasmobranchs are a primarily carnivorous group of vertebrates obtaining little glucose from their diet. They also have very low hepatic glycogen stores (0.5-2% compared to 6-8% in mammals and 2-6% in teleosts) due to the accumulation of lipids in the liver. These lipids are used for buoyancy and to produce ketone bodies, such as acetoacetate and β-hydroxybutyrate, which are the preferred fuel for many elasmobranch tissues making these species much less reliant on glucose than other vertebrates species (Ballantyne, 1997). One exception in elasmobranchs is the rectal gland, a salt secreting organ responsible for eliminating excess salts from the body. Walsh et al. (2006) demonstrated that among a variety of metabolic fuels tested, glucose was the only one able to sustain gland secretion on its own. These findings led us to hypothesise that glucose transporters (GLUTs) would be upregulated in the gland upon activation, for instance following a meal, or exposure to a hypersaline environment. However, a thorough literature search revealed that very little is known about elasmobranch glucose transporters in general. A study by Balmaceda-Aguilera et al. (2012) showed that the GLUT1 protein is expressed in the brain, and there has been speculation about the presence of GLUT2 and the absence of GLUT4 in elasmobranchs (Anderson et al., 2002).

The transporters investigated in the current study are GLUT1-4, which compose class I of the facilitated diffusion glucose transporters (solute-carrier family 2A) and are the best characterised GLUTs in other vertebrates (see reviews by Scheepers et al., 2004; Thorens and Mueckler, 2010; Wood and Trayhurn, 2003; Zhao and Keating, 2007). Briefly, GLUT1 is a ubiquitously expressed transporter that is responsible for the general uptake of glucose by tissues. In mammals, the protein is found in all tissues but is most highly expressed in the brain and in the erythrocytes, and thus it has sometimes been referred to as the erythrocyte-type transporter. The mRNA for GLUT1 is ubiquitously expressed across tissues in all vertebrate groups examined to date (Table 1). GLUT2 is a bidirectional transporter and the protein and mRNA are expressed highly in the liver, intestine, and kidney, and also in the pancreatic β-cells where it acts as a glucose sensor to signal the release of insulin. GLUT3 is a high affinity transporter whose protein is abundant in tissues with high glucose needs, namely the brain and testis, whereas the mRNA is expressed ubiquitously across tissues. GLUT4 is the insulin-responsive transporter which is stored within the cell and transported to the membrane when signalled by insulin. In mammals, the protein and mRNA are expressed highly in the heart, brain, skeletal muscle, and adipose tissue, whereas teleosts also highly express this transporter in the gills and birds are not believed to express this transporter at all, as they rely more on fructose than on glucose and thus require other transporter types, namely GLUT5.

**Table 1.**
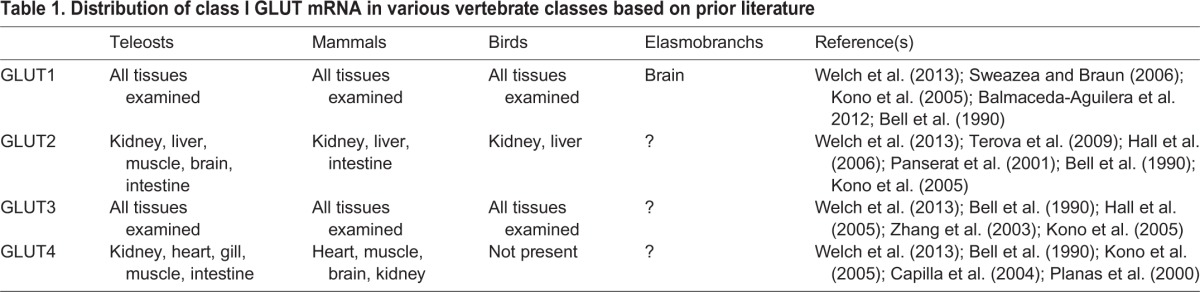
**Distribution of class I GLUT mRNA in various vertebrate classes based on prior literature**

The presence of these four transporters has been confirmed in most vertebrate groups, but is yet to be investigated in the cartilaginous fishes (elasmobranchs and holocephalans). Thus, we used the transcriptomes for three elasmobranchs, the little skate (*Leucoraja erinacea*), the North Pacific spiny dogfish (*Squalus suckleyi*), and the small-spotted catshark (*Scyliorhinus canicula),* and one holocephalan, the elephantfish (*Callorhinchus milii*), to find potential GLUT sequences that could be used to construct the phylogeny of the transporters, as well as to assess which transporters are present in the cartilaginous fishes. We found three putative GLUT sequences in each of all four transcriptomes, but the transporter types differed between the elasmobranchs and the holocephalan. Once identity of the sequences was confirmed, we then used them to determine tissue distribution of each transporter as well as measure their mRNA levels in the rectal gland, liver, muscle, and intestine of fed and fasted spiny dogfish.

## RESULTS

### Phylogenetic analysis and tissue distribution

**Fig. 1.**
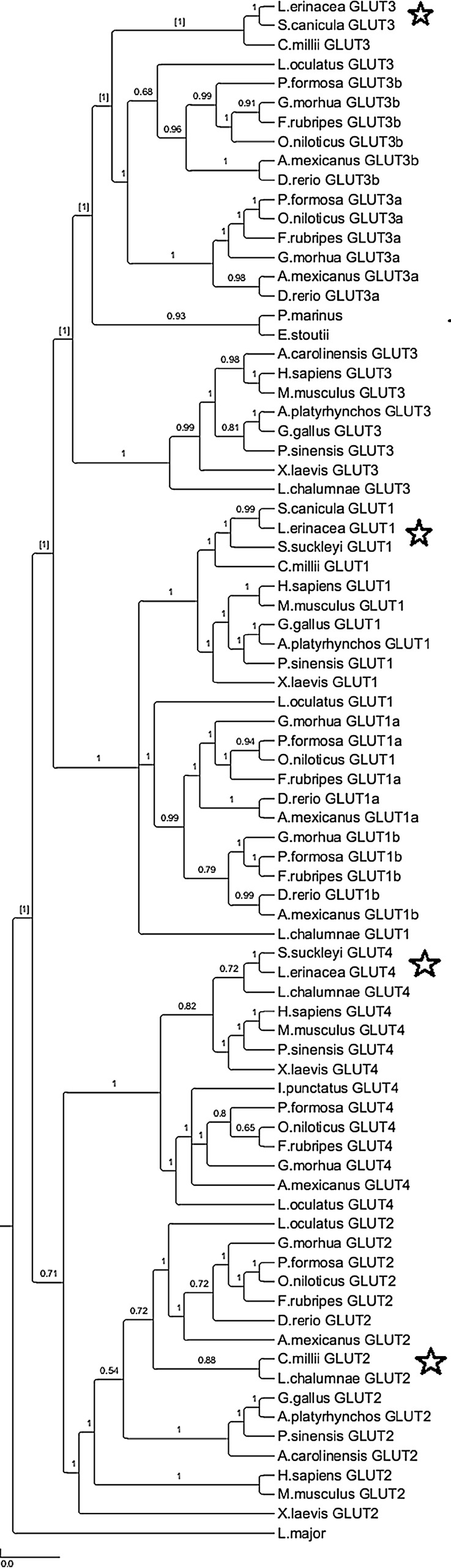
**Phylogeny** **of the glucose transporters in vertebrates using a Bayesian framework.** GLUT sequences were aligned using MUSCLE and the Bayesian phylogeny created using BEAST. Numbers indicate posterior probabilities; stars show the position of the cartilaginous fish on the tree; scale bar represents the mean number of nucleotide substitutions per site.

**Fig. 2.**
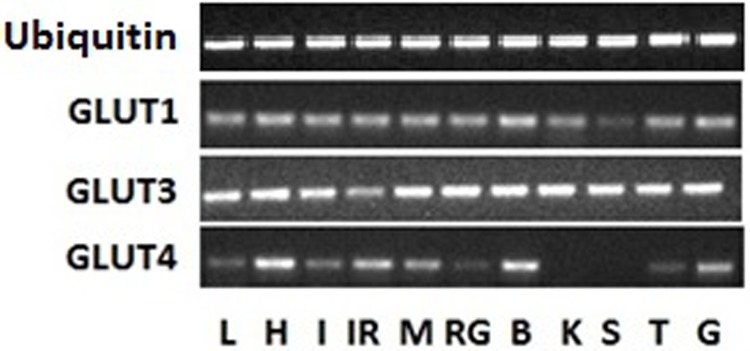
**Representative tissue distribution of ubiquitin and glucose transporter (GLUT) 1, 3 and 4 in *S. suckleyi*.** L, liver; H, heart; I, intestine; IR, interrenal; M, muscle; RG, rectal gland; B, brain; K, kidney; S, spleen; T, testis; G, gill.

Three sequences in the dogfish, little skate, catshark and elephantfish transcriptomes were identified as possible GLUT sequences (see Supplementary information). These were used to recreate a phylogenetic tree which revealed the putative identities of each sequence (Fig. 1). A GLUT1-like sequence was identified in all four transcriptomes. The amino acid sequences were 77-84% identical to human GLUT1 and contained the functional motifs characteristic of class I transporters. A GLUT3-like sequence was also found in all four transcriptomes with amino acid sequences ranging from 60-67% identical to human GLUT3. A GLUT2-like sequence was found solely in the holocephalan transcriptome and the amino acid sequence was 62% identical to human GLUT2. Lastly, a GLUT4-like sequence was found in the dogfish and skate transcriptomes which were 64-69% identical to human GLUT4. The only full sequence belonged to the little skate and it contained the PSLL and TELEY C-terminus motifs (although the latter contained one substitution) and the FQQI N-terminus motif (again, with a single substitution) characteristic of vertebrate GLUT4s. We obtained the majority of the dogfish GLUT4 sequence and this also contained the characteristic C-terminus motifs. We were able to clone GLUT1, 3 and 4 in the dogfish in order to determine in which tissues the mRNA was being expressed (Fig. 2). GLUT1 and 3 showed a ubiquitous expression, with relatively even amounts present in each tissue. GLUT4, however, was slightly more tissue-specific. It was expressed highly in the brain, heart, muscle, gill, and inter-renal gland with lower levels present in the liver and intestine. Attempts were made to isolate a GLUT2-like gene in the dogfish despite the absence of such a sequence. Both regular and consensus primer sets were designed using the holocephalan sequence, teleost sequences, and mammalian sequences but all attempts to isolate the gene were unsuccessful.

### Plasma and tissue analysis

**Fig. 3.**
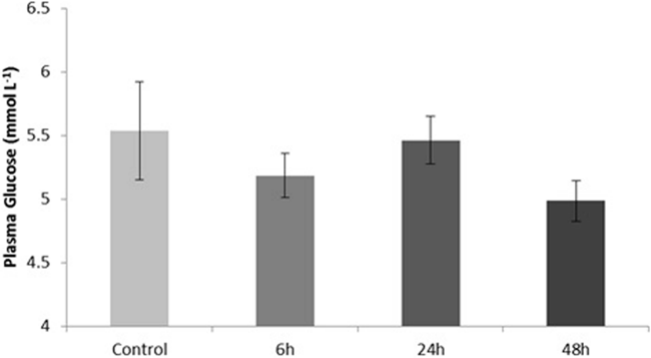
**Plasma glucose in response to feeding in *S. suckleyi*.** Control fish were starved for 7 days. Glucose was measured using the hexokinase method. Data are means±s.e.m (*n*=7).

**Fig. 4.**
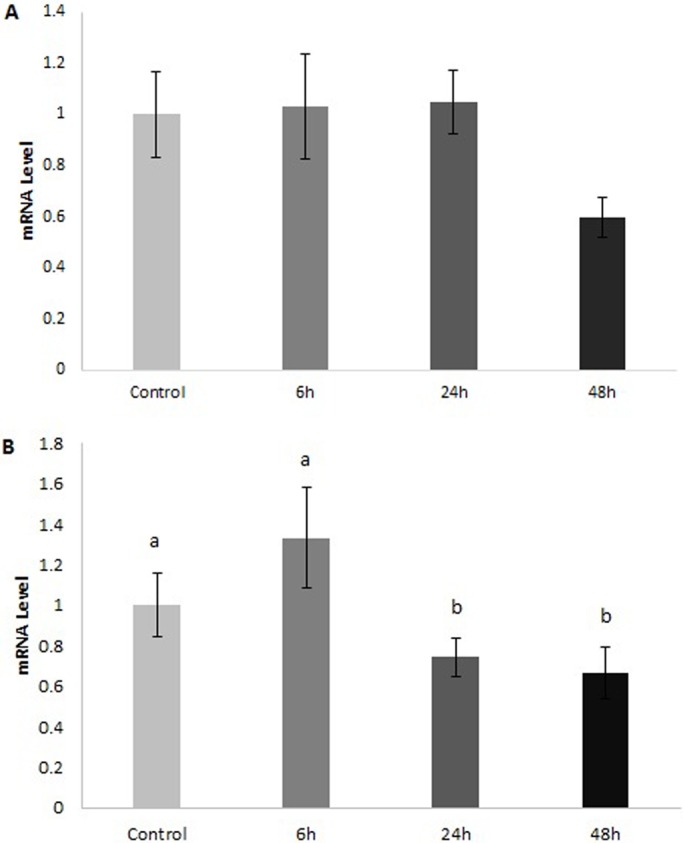
***GLUT1* and *GLUT4* mRNA levels in the rectal gland of *S. suckleyi* at various times post-feeding**. *GLUT1* (A) and *GLUT4* (B) relative mRNA levels determined by quantitative PCR. Control fish were starved for 7 days. Data are means±s.e.m. Different letters indicate significant differences between time points (one-way ANOVA; *n*=7; *P*<0.05).

**Fig. 5.**
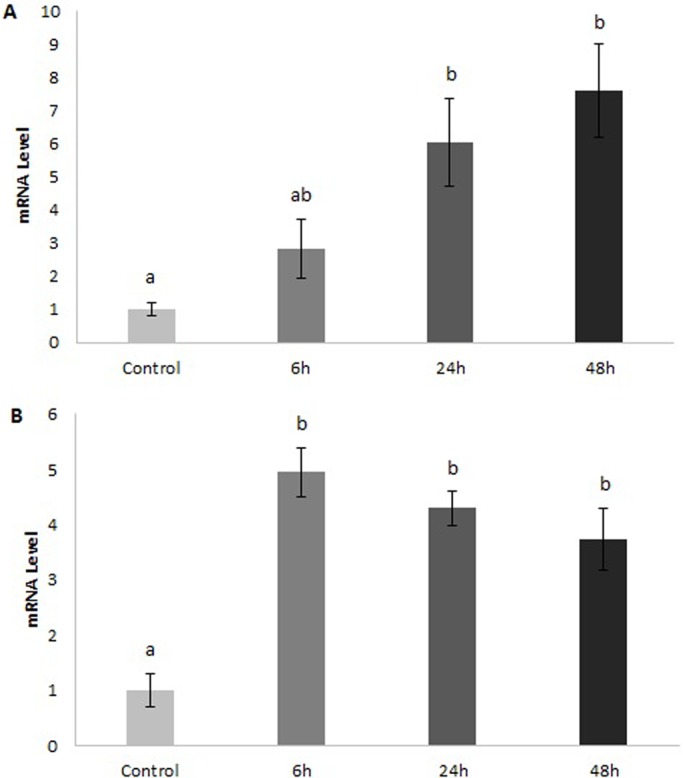
***GLUT4* mRNA levels in the liver and intestine of *S. suckleyi* at various times post-feeding.** Liver (A) and intestine (B) mRNA levels as determined by quantitative PCR. Control fish were fasted for 7 days. Data are means±s.e.m. Different letters indicate significant differences between time points (one-way ANOVA; *n*=7; *P*<0.01).

**Fig. 6.**
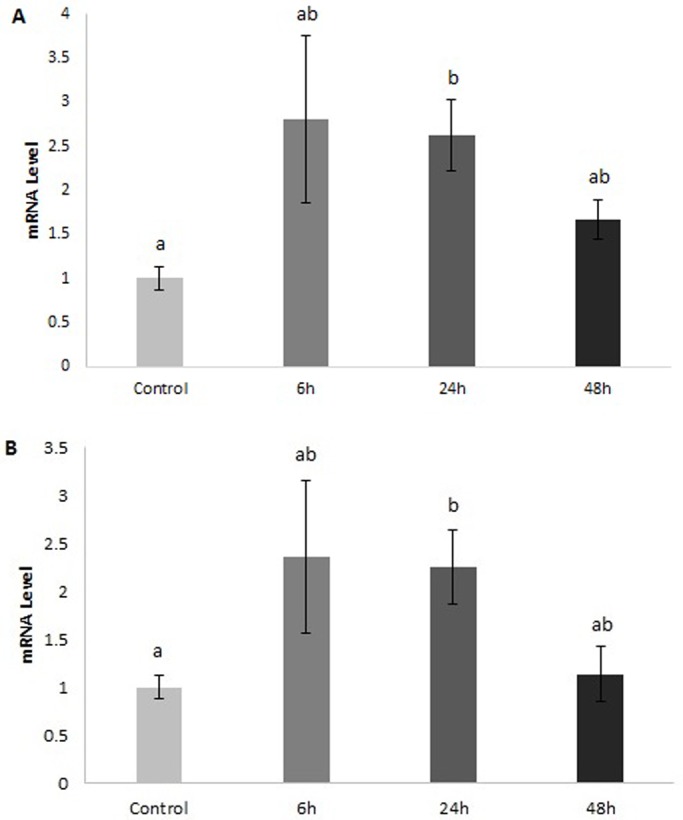
***GLUT1* and *GLUT4* mRNA levels in the white skeletal muscle of *S. suckleyi* at various times post-feeding.** *GLUT1* (A) and *GLUT4* (B) mRNA levels as determined by quantitative PCR. Control fish were starved for 7 days. Data are means±s.e.m. Different letters indicate significant differences between time points (one-way ANOVA; *n*=7; *P*<0.05).

We measured glucose concentrations in the plasma and observed a slight decrease from 5.54±0.39 (mean±s.e.m). in the control fish to 4.98±0.16 at 48 h post-feeding but the change was not significant (Fig. 3). qPCR was then used to measure the expression of *GLUT1*, *3* and *4* in the rectal gland, intestine, liver, and muscle of fed and fasted dogfish. *GLUT1* in the rectal gland showed a trend toward a decrease in mRNA levels at 48 h post-feeding but the change was not significant (Fig. 4A). *GLUT4* on the other hand had significantly lower mRNA levels at both 24 and 48 h post-feeding (Fig. 4B). In the liver and intestine, *GLUT1* mRNA levels were not significantly different across time points. *GLUT4*, however, was significantly higher at 24 and 48 h post-feeding in the liver relative to the fasted fish (Fig. 5A). In the intestine, there was a 5-fold increase in *GLUT4* mRNA levels by 6 h post-feeding and this was maintained through the 24 and 48 h time points (Fig. 5B). In the muscle, we observed a significant increase in both *GLUT1* and *GLUT4* mRNA by 24 h post-feeding and this returned to fasted levels by 48 h (Fig. 6). *GLUT3* did not change significantly in any of the tissues examined.

## DISCUSSION

In this study, we sought to identify class I GLUTs from selected elasmobranch and holocephalan species as they have not been thoroughly investigated in cartilaginous fish, but are likely important transporters, particularly in the rectal gland which requires glucose as a metabolic fuel. Three potential GLUT sequences were present in the transcriptomes of both the elasmobranchs and holocephalans but the identities of the transporters differed. Both groups possess GLUT1- and 3-like sequences, but we were unable to retrieve a GLUT2 sequence for the elasmobranchs and a GLUT4 sequence for the holocephalan, and attempts to isolate these transporters were unsuccessful. This does not provide definitive evidence against the presence of GLUT2 in the elasmobranchs and GLUT4 in the holocephalans as the transporter may have diverged to the point that it is unrecognizable or could have become a pseudogene. However, it may speak to differing selection pressures in the two taxa and reflect differences in the energy metabolism of these fish. For instance, since glucose is of limited importance in these species they could have either lost or developed a new function for one of the transporters. Given that GLUTs 2 and 4 appear to be duplications of one another (as suggested by the phylogeny), it is not implausible that GLUT2 in the holocephalans and GLUT4 in the elasmobranchs took on both roles while the ‘lost’ transporter was converted into a protein that would be of more use to them. It is interesting to note that GLUT4 appears to also have been lost in birds. This loss is believed to be the cause of their apparent insulin resistance and the higher plasma levels of glucose relative to other vertebrates (reviewed by Braun and Sweazea, 2008). It would be interesting to see if this is also the case in holocephalans.

The transporter phylogeny presents GLUTs 1 and 3 branching off into one clade while GLUTs 2 and 4 branch off into a separate clade, supporting the 2R genome duplication hypothesis (Sidow, 1996). However, only one GLUT sequence is known for the cyclostomes. We believe it is likely that they possess a GLUT4-like transporter as both the hagfish and lamprey are responsive to insulin (Emdin, 1982; Larsen, 1976), but the loss or functional divergence of a second transporter is also possible. In addition, most teleosts only possess one each of GLUTs 2 and 4, whereas there are both ‘a’ and ‘b’ forms for GLUT1 and 3, suggesting that duplicates of these genes may have been lost due to a lack of necessity. Interestingly though, two GLUT4 isoforms have been reported in the muscle of Atlantic salmon (*Salmo salar*; Menoyo et al., 2006) which may reflect the tetraploid nature of the salmonids. GLUT2 and 4 are very tissue-specific and have definitive roles, whereas GLUT1 and 3 are ubiquitously expressed and play a more general role in glucose uptake. Thus, the maintenance of two forms of GLUT1 and 3 may allow for greater versatility across tissues and under varying conditions. For instance, studies have shown changes in the expression of these two transporters in response to hypoxia and/or changes in salinity in various tissues (Balmaceda-Aguilera et al., 2012; Hall et al., 2005; Zhang et al., 2003). Unfortunately, all three studies only measured one of the GLUT1 or 3 transcripts so we cannot determine whether the two forms have varying roles or even be sure that the same transcript was picked up in each tissue. This would be an interesting avenue for future studies.

In addition to reconstructing the phylogeny, we examined the expression of *GLUT1*, *3* and *4* in various dogfish tissues and observed some interesting patterns. *GLUT1* and *GLUT3* mRNA was present in all tissues examined which is consistent with what has been observed in a number of other vertebrates. Conversely, *GLUT4* was much more tissue-specific. The highest mRNA levels were found in the heart, brain, skeletal muscle, and gill, which again is similar to the expression pattern seen in other vertebrates. Interestingly though, *GLUT4* mRNA was also expressed in the liver and intestine, tissues that express *GLUT2* in most other vertebrates. This observation reinforces the idea that *GLUT2* may have been lost in elasmobranchs due to a lack of necessity (limited glucose intake) and its role taken over by *GLUT4*. Further investigation on the characteristics of this transporter in the different tissues is warranted. One interesting observation was the lack of *GLUT4* mRNA in the elasmobranch kidney as both *GLUT2* and *GLUT4* are expressed in the kidney in mammals and teleosts. This lack of expression may reflect the limited importance of the elasmobranch kidney in salt secretion due to the presence of the rectal gland.

The final aspect of this study was the measurement of GLUT mRNA levels in specific dogfish tissues in response to feeding and fasting. The four tissues investigated were the rectal gland, liver, muscle, and intestine. In the rectal gland, we saw that the mRNA levels for both *GLUT1* and *4* decreased post-feeding, although we only found significance for *GLUT4*. Prior to degradation, mRNAs are deadenylated (removal of the poly-A tail) and decapped (removal of the 5′ cap). However, previous studies have described the translational repression and storage of mRNAs that are yet to be decapped and this can occur during times of stress (reviewed by Coller and Parker, 2004). Previous transcriptome work in the rectal gland by Deck et al. (2013) illustrated that mRNA was being stored in fasted glands for a number of genes, most notably Na^+^/K^+^-ATPase, which we know has lower protein abundance and enzyme activities in starved glands (Dowd et al., 2008; Walsh et al., 2006). Thus it is possible that during fasting, the rectal gland is storing the mRNA of genes that are essential for immediate activation of the gland upon feeding. We were unable to measure protein levels for GLUTs in the dogfish as the mammalian antibodies available failed to bind but since we know that the gland is glucose-dependent, it would not be surprising that it would store mRNA for the GLUTs so as to allow for rapid protein synthesis upon activation.

In the intestine, we observed a significant increase in *GLUT4* mRNA at 6 h post-feeding and expression remained elevated through the 48 h. The intestine is one of the first tissues to be exposed to the nutrients from the incoming meal, so it is appropriate that it should rapidly upregulate essential transporters. It would be interesting to determine exactly what causes this upregulation as the meal has very little glucose. We also observed a significant increase in *GLUT4* mRNA in the liver, only this increase was slightly delayed relative to the intestine, not being elevated until 24 h post-feeding. However, the liver would not be exposed to the incoming nutrients as early as the intestine, and given the slow digestion rate of most elasmobranchs, the upregulation at 24 h appears to be an appropriate response and this could explain the slight decrease in plasma glucose that was observed. What is interesting about the expression patterns observed in the intestine and liver is that this is what would be expected of GLUT2 in other vertebrates, possibly providing more evidence for the loss of that transporter in these fish. Lastly, we observed significant increases in both GLUT1 and 4 in the muscle at 24 h post-feeding and this returned to fasting levels by 48 h. In brown trout (*Salmo trutta*) muscle, fasting has been shown to decrease GLUT4 levels whereas insulin increases GLUT4 levels (Diaz et al., 2007). Furthermore, insulin has been shown to regulate the expression of both GLUT1 and 4 in the muscle of rainbow trout (Diaz et al., 2009). Amino acids are insulinotropic substances in most vertebrates and are believed to be more insulinotropic than glucose in elasmobranchs due to their carnivorous nature. Thus, it is likely that these increases are due to the actions of insulin, but future studies investigating the effects of insulin infusion on GLUT levels in elasmobranch muscle are warranted.

This is only the second report of GLUTs in elasmobranchs (the first being the study by Balmaceda-Aguilera et al., 2012 that established the presence of GLUT1 in the shark brain) and the first study to identify and show tissue distribution for multiple GLUTs. Furthermore, the basal position of the elasmobranchs on the vertebrate tree has allowed us to provide valuable insight into the evolution of these transporters, although much work remains to be done. Future studies should aim to characterise the function of each transporter and determine what conditions modulate their expression in the different tissues.

## MATERIALS AND METHODS

### Animals and feeding

Adult male North Pacific spiny dogfish (*Squalus suckleyi*) ranging in weight from 1.5-2.5 kg were obtained by angling from Barkley Sound (BC, Canada) and transferred to Bamfield Marine Sciences Centre in August 2013 and June 2014. The sharks were maintained in a 155,000 litres circular tank with running seawater and aeration. Following a two week acclimation period, dogfish were fed a ration (2.5% body weight) of frozen hake every third day. One group of fish was transferred to a separate tank following a meal where they were fasted for seven days, while other groups were fasted for seven days, fed, and then euthanized at 6 h, 24 h, or 48 h post-feeding (*N*=7) by MS-222 overdose (1 g l^−1^) and subsequent cervical dislocation. Fish were randomly allotted to each experimental group. Plasma, rectal gland, intestine, liver, and muscle samples were frozen in liquid nitrogen and then stored at −80°C for further analysis. Plasma glucose was measured using the Infinity Hexokinase Reagent according to the manufacturer's instructions (Thermo Scientific). All experiments were conducted in accordance with guidelines of the Canadian Council of Animal Care, and under approvals from the Animal Care Committees of the University of Ottawa and Bamfield Marine Sciences Centre.

### Phylogenetic analysis

Access to the skate, elephantfish, and catshark transcriptomes was gained online through SkateBase (http://skatebase.org; Wyffels et al., 2014) (see Table 2 for Contig numbers), whereas the dogfish transcriptome was generously provided to us by Dr Greg Goss (University of Alberta, Canada). Mammalian and teleost sequences obtained from Ensembl (http://www.ensembl.org) were used to search each transcriptome (blastn) for potential elasmobranch and holocephalan GLUT sequences. These were then aligned with known GLUT sequences from other species using MUSCLE (Edgar, 2004) and the appropriate nucleotide substitution model (GTR +G +I) identified using jModelTest (Darriba et al., 2012; Guindon and Gascuel, 2003). A phylogeny was then constructed using a Bayesian framework (BEAST; Drummond et al., 2012). The analysis was run for ten million generations with a thinning of 1000 and the first 2500 trees were discarded as burn-in.

**Table 2.**
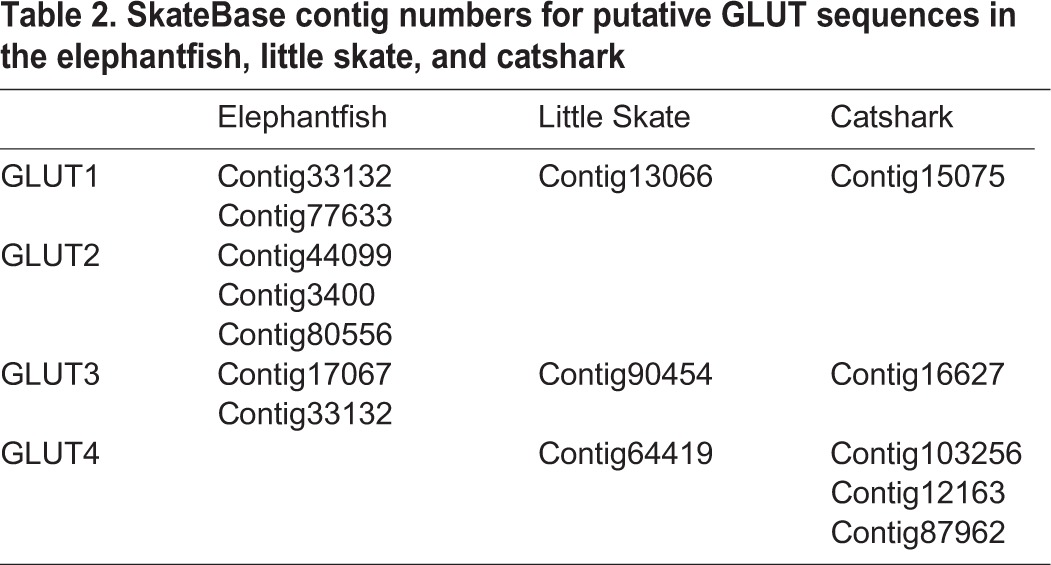
**SkateBase contig numbers for putative GLUT sequences in the elephantfish, little skate, and catshark**

### Tissue distribution

Total RNA was extracted using Trizol reagent (Invitrogen) according to the manufacturer's instructions, treated with DNase I (Invitrogen), and used as a template for reverse transcription (RT). The RT reactions combined 2 μg RNA (quantified spectrometrically by NanoDrop) with 375 ng random hexamers [Integrated DNA Technologies (IDT)], 125 ng oligo dTs (IDT), and dNTPs (Invitrogen; 0.5 mM final concentration) and the mixture was incubated at 65°C for 5 min. The reaction was then chilled on ice for 5 min and first-strand reaction buffer (Invitrogen; 1× final concentration), dithiothreitol (5 mM final concentration), RNase Out (Invitrogen; 0.5 μl), and nuclease-free water (0.5 μl) were added. The mixture was incubated at 42°C for 2 min and then combined with Superscript II reverse transcriptase (Invitrogen; 1 μl) to a total volume of 20 μl. Lastly, the reaction was incubated at 42°C for 50 min and inactivated at 72°C for 15 min.

Following RT, the cDNA was amplified by adding 1 μl of diluted template (1:5) to a 25 μl total reaction volume containing PCR reaction buffer (Denville Scientific; 1× final concentration), dNTPs (Invitrogen; 0.2 mM final concentration), 0.15 μl Choice Taq (Denville Scientific), and gene-specific sense and antisense primers (IDT; 0.2 μM final concentrations). Primers for each gene were derived from sequences found in the dogfish transcriptome. The cycling parameters were 94°C for 30 s followed by 40 cycles of 94°C for 30 s, 60°C for 30 s, and 72°C for 30 s and concluding with 15 min at 72°C. The products were then visualized on a 1.5% agarose gel.

### qPCR

For real-time PCR, the cDNA template was prepared as described above and 1 μl of the diluted template was added to a 12.5 μl total reaction volume containing 6.25 μl Rotor-Gene SYBR Green PCR master mix (Qiagen) and 200 nM each gene-specific sense and antisense primer (Table 3). We used Norma-Gene (Heckmann et al., 2011) to normalise the rectal gland samples as both EF1α and ubiquitin changed with our treatments in this tissue. Relative mRNA levels for intestine, muscle, and liver were normalised to *S. suckleyi* ubiquitin levels. Norma-Gene could not be used because not enough genes were run in these tissues but ubiquitin levels appeared to be constant across our treatments (i.e. levels were not statistically different from one another). Fluorescence was detected using a Rotor-Gene Q Real Time PCR cycler (Qiagen) with the following cycling parameters: 95°C for 5 min followed by 40 cycles of 95°C for 5 s and 60°C for 10 s. All reactions were run in duplicate and relative mRNA levels were calculated using the delta Ct method. One-way ANOVAs and Holm–Sidak post-hoc tests were used to compare mRNA levels between treatments. When the equal variance test failed, a one-way ANOVA on Ranks with a Dunn's post-hoc test was conducted.

**Table 3.**
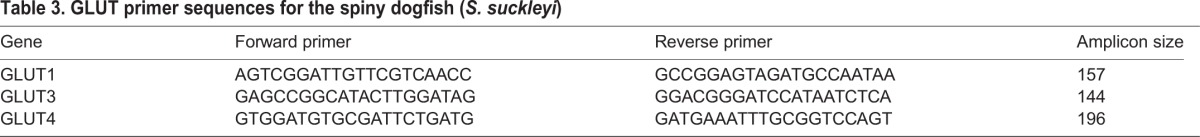
**GLUT primer sequences for the spiny dogfish (*S. suckleyi*)**
